# Preoperative and Postoperative Change in Patient-Reported Health-Related Quality of Life Outcomes in Breast Cancer Surgery Patients Across Surgical Modalities: A Prospective Study

**DOI:** 10.3390/cancers17091409

**Published:** 2025-04-23

**Authors:** Claire Liu, Aidan Beresford, Maria Saleeb, Guiping Liu, Trafford Crump, Rebecca Warburton, Jin-Si Pao, Carol K. Dingee, Amy Bazzarelli, Jason M. Sutherland, Elaine C. McKevitt

**Affiliations:** 1 Department of Surgery, Faculty of Medicine, University of British Columbia, Vancouver, BC V5Z 1M9, Canada; 2 Centre for Health Services and Policy Research, University of British Columbia, Vancouver, BC V6T 1Z3, Canadajason.sutherland@ubc.ca (J.M.S.); 3 Faculty of Medicine and Health Sciences, McGill University, Montreal, QC H4A 3J1, Canada; 4 Providence Breast Centre, Mount Saint Joseph Hospital, 3080 Prince Edward Street, Vancouver, BC V5T 3N4, Canada

**Keywords:** patient-reported outcomes, health-related quality of life, breast cancer surgery, breast cancer survivorship

## Abstract

**Background**: This study compared the change in pre- and postoperative health-related quality of life (HRQoL) among breast cancer patients undergoing breast-conserving surgery (BCS), total mastectomy no reconstruction (TMNR), and total mastectomy immediate breast reconstruction (MIBR). Patient factors associated with postoperative anxiety and depression were also identified. **Methods**: This prospective cohort study enrolled breast cancer patients between September 2017 and August 2020. HRQoL changes from preoperative to six months postoperative were compared using patient-reported outcome tools assessing anxiety, depression, pain, perceived health, breast satisfaction, psychosocial, physical, and sexual well-being and analyzed with ANOVA and linear regression. **Results**: A total of 471 patients completed preoperative and postoperative surveys (BCS: 313, TMNR: 60, MIBR: 98). Postoperative anxiety decreased across all modalities, with MIBR showing the greatest reduction (*p* = 0.03), though still exhibiting the highest postoperative anxiety (*p* = 0.05). Depression and perceived health scores showed no significant difference in change across modalities (*p* = 0.15, *p* = 0.48). MIBR patients showed the greatest increase in pain (*p* = 0.05) and the highest postoperative pain scores (*p* = 0.04). All three modalities showed a clinically significant decline in physical and sexual well-being. TMNR and MIBR had additional reductions in breast satisfaction, with TMNR also showing a decline in psychosocial well-being. Absolute postoperative scores for breast satisfaction, psychosocial, physical, and sexual well-being remained highest in BCS compared to TMNR and MIBR (*p* < 0.01, for each domain). In multivariable regression analysis, postoperative depression and anxiety scores did not differ between surgical modalities, but younger age was significantly associated with higher postoperative depression, pain and anxiety (*p* < 0.01), and adjuvant chemotherapy with higher postoperative depression (*p* < 0.01). **Conclusions**: BCS may have better overall HRQoL outcomes, specifically in breast satisfaction, psychosocial, physical, and sexual well-being, compared to TMNR and MIBR. Additionally, younger age, rather than surgical modality, was found to be associated with higher postoperative depression, pain, and anxiety scores.

## Simple Summary

Although breast-conserving surgery has been shown to have excellent oncologic outcomes, some patients opt for treatment with mastectomy, with or without reconstruction, which is a more invasive surgical procedure. Understanding pre- and postoperative health-related quality of life for these procedures will help to inform physicians and patients when choosing breast cancer treatment. Patients undergoing breast cancer surgery at our center were invited to complete health-related quality of life surveys pre- and postoperatively to assess anxiety; depression; pain; perceived health; breast satisfaction; and psychosocial, physical, and sexual well-being. The results were compared between the three surgical approaches. A total of 471 patients completed both sets of surveys. BCS may have better overall HRQoL outcomes, specifically in breast satisfaction, psychosocial, physical, and sexual well-being, compared to mastectomy. Additionally, younger age, rather than surgical modality, was found to be associated with higher postoperative depression, pain, and anxiety scores.

## 1. Introduction

The psychological impact of receiving a breast cancer diagnosis for many patients is considerable, with women reporting poor sleep, depression, and anxiety before their surgery [1,2,3]. Although breast cancer surgery is expected to improve survival, breast cancer survivors had worse health-related quality of life (HRQoL) up to ten years following their diagnosis than people who did not have breast cancer [4].

Operative treatment is offered to most breast cancer patients, with options including breast-conserving surgery (BCS) and total mastectomy. Although many patients are suitable candidates for breast-conserving surgery, some opt for mastectomy [5,6,7,8]. Despite this preference, mastectomy is a more invasive procedure that has not been shown to improve cancer outcomes and may be associated with poorer postoperative HRQoL for patients [9,10,11,12]. A meta-analysis found that patients who received BCS reported better HRQoL in several domains, including social, emotional, and overall health, compared with those who received mastectomy [12]. Few existing studies have compared the preoperative to postoperative change in HRQoL between different treatment modalities of BCS, total mastectomy with no reconstruction (TMNR), and mastectomy with immediate breast reconstruction (MIBR). Additionally, most of these studies do not account for patient characteristics such as age, comorbidities, or socioeconomic factors. These factors are relevant as they may be independently predictive of a patient’s reported HRQoL [6].

In this study, we measured the change in patient-reported HRQoL between the preoperative and six-month postoperative period in a cohort of women with breast cancer, directly comparing outcomes from BCS, TMNR, and MIBR. Additionally, we performed a multivariable regression analysis to identify patient factors associated with postoperative anxiety and depression. This study contributes unique and timely knowledge pertaining to the association between patients’ self-reported health and well-being outcomes with BCS, TMNR, and MIBR.

## 2. Materials and Methods

### 2.1. Patient Recruitment and Study Design

Consecutive patients scheduled for mastectomy (i.e., TMNR and MIBR) or BCS between September 2017 and August 2020 were prospectively recruited from a Canadian breast cancer referral center. Patients were eligible for this study if they were able to respond to questionnaires in English, were 18 years or older, and were community-dwelling. Patients who were eligible for participation were contacted by phone and asked to complete a survey including demographic information and several patient-reported outcome measures (PROMs) that measured their mental and physical health. Based on participant preference, PROMs could be answered through a mailed survey package or securely online. Participants completed their preoperative PROMs shortly after consenting before their surgery, and they completed the same PROMs six months postoperatively.

The study protocol was approved by the University of British Columbia Clinical Research Ethics Board. Patient information was de-identified and coded.

### 2.2. Patient Reported Outcome Measures

#### 2.2.1. Patient-Health Questionnaire-9 (PHQ-9)

The PHQ-9 measures symptoms of depression through nine items, with each item ranging from 0 (not bothered by symptoms) to 3 (bothered nearly every day by symptoms). Items are totaled to provide a score out of 27, with higher scores reflecting worse symptoms [13]. The PHQ-9 is a widely validated measure that is valid and reliable (Cronbach’s alpha = 0.84) among cancer patients [14].

#### 2.2.2. Generalized Anxiety Disorder-7 (GAD-7)

The GAD-7 measures symptoms of anxiety through seven items that range from 0 (not at all) to 3 (nearly every day). Item scores are summed to provide a score with maximum of 21 [15]. The GAD-7 is a reliable and valid measure for evaluating anxiety in cancer patients [16]. Cronbach’s alpha in this study = 0.91.

#### 2.2.3. Pain Intensity (P), Interference with Enjoyment of Life (E), and Interference with General Activity (G), PEG

The PEG is a brief three-item questionnaire that evaluates pain intensity and interference with quality of life [17]. Each item ranges from 0 (no pain or interference) to 10 (as bad as you can imagine), and items are summed. The PEG has shown reliability (Cronbach’s alpha = 0.73) and validity in primary care patients [18].

#### 2.2.4. EuroQoL EQ-5D-5L VAS

EQ-5D-5L VAS is a general health PROM that assesses mobility, self-care, usual activities, pain/discomfort, and anxiety/depression [19]. The measure also includes a visual analogue scale (VAS) that asks patients to rate their present health from 0 (worst health you can imagine) to 100 (best health you can imagine) [19]. EQ-5D-5L VAS is a widely used measure for many patient cohorts, including cancer patients, and has been reported to be valid and reliable [20].

#### 2.2.5. BREAST-Q

BREAST-Q is a validated breast cancer-specific PROM. The questionnaire is presented according to surgical procedure type (BCS, mastectomy, and breast reconstruction) and focuses on two domains of HRQoL, including overall quality of life and patient satisfaction [21,22,23]. The questionnaire consists of domains that measure patient expectations and experiences with care and their psychosocial, sexual, and physical well-being. Items in each domain are scored to derive a total domain score ranging from 0 (none, dissatisfied, and disagree) to 100 (all of the time, satisfied, and agree) [22]. In this study, we used the core BREAST-Q module that consists of four domains assessing satisfaction with breasts, psychosocial, sexual, and physical chest well-being.

### 2.3. Sociodemographic Characteristics and Clinical Factors

To measure aspects of participants’ socioeconomic status (SES), the Canadian Index of Multiple Deprivation (CIMD) was used, which is made available by Statistics Canada and derived independently of this study. The indices represent neighborhood-level measures, representing approximately 140 households, and have been found to correlate with individual socioeconomic status and multiple health outcomes [24,25,26]. The CIMD ethnocultural composition and situational vulnerability index were linked with the participants’ postal code. The ethnocultural composition variable measured the proportion of households that self-identified as a visible minority, were foreign-born, had no knowledge of either official language (English or French), or were recent immigrants (arrived in the 5 years prior to census data collection). The situational vulnerability variable measures the proportion of households that are low-income, aged 25–64 years without a high school diploma, are single-parent families, self-identified as Aboriginal, and the proportion of dwellings needing major repair in the neighborhood [27]. Each measure was reported in quintiles ranging from 1 (least of that index) to 5 (most of that index).

Patients’ PROMs were linked with their hospital discharge summaries to identify other demographic and disease characteristics, such as age and comorbidities. Using participants’ comorbidities, the Charlson Comorbidity Index (CCI) score was calculated to aid in quantifying the burden of illness a participant may be experiencing [28]. A higher CCI score is associated with higher burden of illness and greater mortality risk.

### 2.4. Statistical Analysis

The frequency distribution of age, CCI, and SES of the overall sample were calculated. These variables were categorized to display the range of participants. Analyses were presented for the overall sample and stratified by BCS, TMNR, and MIBR. The PROM scores of patients before and six months after surgery were categorized by participants receiving BCS and those receiving TMNR and MIBR.

Differences in means of continuous variables were compared using Student *t*-tests, and differences in frequency counts for categorical variables were compared using ANOVA and Pearson chi-square tests. Multivariable linear regression was used to measure the association between participant demographic/baseline patient characteristics (age, surgical modality, sociodemographic variables, and CCI), with participants’ six-month postoperative PHQ-9, GAD-7 and PEG scores. Significance was set at *p* < 0.05. All statistical analyses were conducted using SAS/STAT software, Version 9.4.

## 3. Results

### 3.1. Sample Characteristics

Between September 2017 and August 2020, 805 eligible breast cancer patients scheduled to undergo breast cancer surgery were contacted, and 671 patients were recruited and completed the preoperative PROMs surveys. At six months follow-up, 471 (70%) of participants completed the postoperative surveys. Patients who completed the surveys were, on average, 2.4 years older compared to those who did not complete the surveys. There were no differences in SES or CCI scores between participants and nonparticipants.

The majority of participants underwent BCS (n = 313, 66.5%), and of those who had total mastectomy, a larger proportion had MIBR (n = 98, 62.0%). The majority of MIBR patients had an implant-based reconstruction with bioprosthesis (85.7%), with 14 patients (14.3%) having autologous reconstruction (DIEP, TRAM, Latissimus flaps). The median age of participants was 59 years, and the majority were from less situationally vulnerable quintiles of the population and more ethnically diverse neighborhoods (Table 1). Participants who underwent TMNR were more likely to have a higher CCI compared to those who underwent BCS or MIBR (*p* < 0.01). Patients who underwent BCS had significantly lower rates of neoadjuvant (*p* < 0.01) and adjuvant chemotherapy (*p* < 0.01). As expected, BCS patients had significantly higher rates of radiation (82.43%, *p* < 0.01). However, nearly half of mastectomy patients also received adjuvant radiation, including 48.33% of TMNR and 42.86% of MIBR patients. There was no difference in the rates of hormone therapy across surgical modalities (*p* = 0.63).

### 3.2. PHQ-9, GAD-7, PEG, and EQ-5D-5L VAS Outcomes

**Figure 1:**
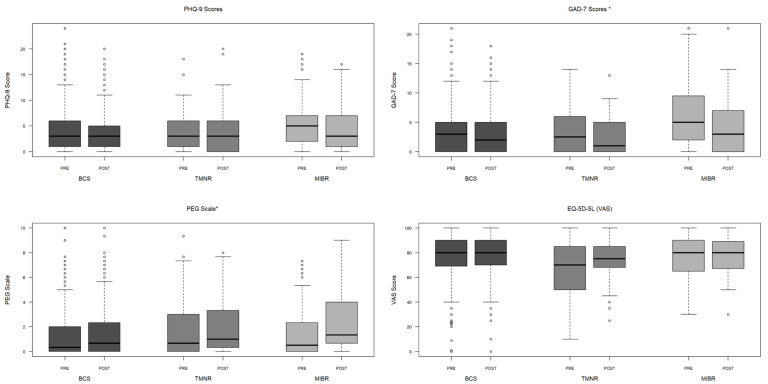
Change in preoperative and 6-month postoperative depression (PHQ-9), anxiety (GAD-7), pain (PEG), and patient-perceived general health (EQ-5D-5L VAS) scores compared across breast-conserving surgery (BCS), total mastectomy with no reconstruction (TMNR), and total mastectomy with immediate breast reconstruction (MIBR). Mean change in GAD-7 and PEG scores when comparing the surgical modalities shows significance (* *p* = 0.03 and *p* = 0.0499, respectively) (see Appendix A Table A1, which includes the summary variables of the figure).

Figure 1 illustrates the change in pre- and postoperative PROM scores for depression (PHQ-9), anxiety (GAD-7), pain (PEG), and patient perceived general health (EQ-5D-5L VAS) stratified by operative procedure (see Appendix A Table A1 for more detail). There was a significant difference in the pre- and postoperative changes in anxiety and borderline significant (*p* = 0.0499) changes in pain scores across the three surgical modalities. There was a decrease in anxiety scores postoperatively across all operative modalities, with the most substantial decrease of 2.43 points observed after MIBR compared to TMNR (−1.61 points) and BCS (−0.91 points) (*p* = 0.03). However, despite this reduction, postoperative anxiety scores remained highest for the MIBR group. Additionally, participants who underwent MIBR reported the highest postoperative pain scores (mean 2.29, SD 2.33) compared to TMNR (mean 1.98, SD 2.21) and BCS (mean 1.65, SD 2.25) (*p* = 0.04) and experienced the largest increase in pain of 0.79 points compared to TMNR (+0.02 points) and BCS (+0.22 points) (*p* = 0.0499). The pre- and postoperative changes in depression and patient-perceived general health scores were similar across the three surgical modalities (*p* = 0.15 and *p* = 0.48, respectively).

### 3.3. BREAST-Q Outcomes

**Figure 2:**
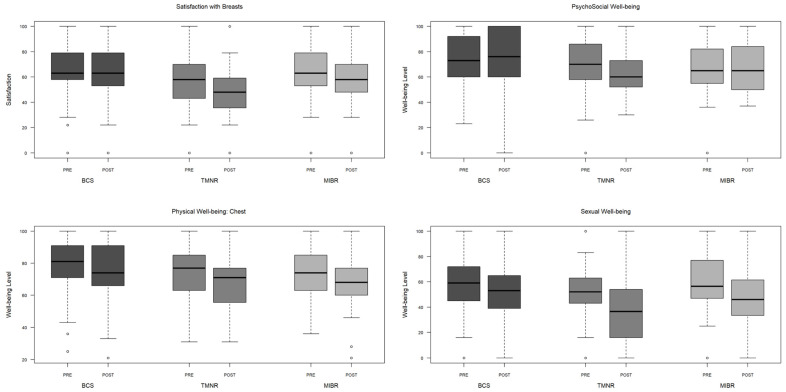
Preoperative and 6-month postoperative BREAST-Q domain scores in breast-conserving surgery (BCS), total mastectomy with no reconstruction (TMNR), and total mastectomy with immediate breast reconstruction (MIBR). Higher scores represent more/higher self-reported well-being (see Appendix A Table A1, which includes the summary variables of the figure).

Overall, TMNR had the poorest BREAST-Q outcomes, as there was a clinically significant [23] decline in all four BREAST-Q domains following TMNR (satisfaction: −7.60, psychosocial: −6.51, physical: −6.06, sexual: −9.41) (Figure 2 and Appendix A Table A1). There was a clinically significant decline in breast satisfaction (−6.33) and physical (−6.38) and sexual well-being (−10.65) following MIBR and a clinically significant decline in only physical (−4.24) and sexual well-being (−5.68) following BCS. Despite these clinically significant changes in pre- to postoperative scores within each surgical modality, there were no statistically significant differences in the pre- to postoperative change in scores between the surgical modalities. Nevertheless, when examining absolute postoperative scores, BCS had significantly higher scores across all four BREAST-Q domains compared to TMNR and MIBR (*p* < 0.01 for each domain; see Appendix A Table A2).

### 3.4. Predictor Effects on Trends in Postoperative Depression, Anxiety, and Pain Scores

Multivariable linear regression measured postoperative PHQ-9, GAD-7, and PEG score and assessed the associations of various patient factors, including age, surgical modality, sociodemographic variables, CCI, and adjuvant therapies (Table 2, Appendix B). Younger age was found to be associated with higher postoperative depression, anxiety, and pain scores (*p* < 0.01), and adjuvant chemotherapy was associated with higher postoperative depression scores (*p* < 0.01). Patients from more vulnerable neighborhoods reported higher pain scores, but neither breast nor axillary procedure was associated with higher pain scores.

## 4. Discussion

In this prospective study of breast cancer surgery patients, we evaluated changes in preoperative and postoperative HRQoL across the surgical modalities BCS, TMNR, and MIBR. Significant differences were observed in anxiety and pain, with MIBR showing the highest values in both. Clinically significant declines were noted across the four BREAST-Q domains within each modality, with TMNR experiencing declines in all four, while BCS had declines in only physical and sexual well-being. Additionally, BCS had significantly higher absolute postoperative scores across all BREAST-Q domains compared to TMNR and MIBR. Our study is unique in that it is the largest longitudinal prospective study comparing the change in preoperative and postoperative HRQoL measures between the breast cancer surgery modalities BCS, TMNR, and MIBR. Additionally, most existing studies investigating patient-reported outcomes in breast cancer patients focus solely on postoperative outcomes [29,30,31], while our study shows the importance of understanding the postoperative PROMs in the context of baseline preoperative values.

The absence of standardized PROMs for breast cancer patients across the literature presents challenges in comparing results. Although BREAST-Q is commonly used in breast cancer-specific PROM studies, conflicting results persist. Two existing single-center prospective cohort studies that compared the preoperative and postoperative changes in breast cancer surgery patient-reported outcomes between BCS, TMNR, and MIBR reported some differing trends from those found in our study.

The first study, conducted by Retrouvey et al. (2019), assessed PROM scores at baseline (preoperative), 6 months, and 12 months postoperatively using the same four BREAST-Q items as our study, but instead used the Hospital Anxiety and Depression Scale (HADS) in evaluating anxiety and depression [32]. Retrouvey et al. found that breast satisfaction and psychosocial well-being scores increased from baseline after BCS at 6 and 12 months, with BCS having the highest postoperative scores across all four BREAST-Q items, followed by MIBR. Although we found similar overall postoperative scores for breast satisfaction with BCS, we did not see the increase shown in Retrouvey’s cohort, and we found a clinically significant decrease in breast satisfaction for our MIBR group. In Retrouvey’s cohort, TMNR had a decrease, and the lowest breast satisfaction, psychosocial well-being, and chest physical well-being scores postoperatively at 6 and 12 months, similar to our findings. They found no significant difference in the mean anxiety and depression scores based on the HADS scale across the surgical modalities preoperatively and at 12 months, whereas we found no difference in depression (*p* = 0.15) but a difference in anxiety score changes (*p* = 0.03) across surgical modalities.

A second study by Devarakonda et al. (2023) assessed only the psychosocial well-being item of the BREAST-Q at baseline (preoperative), 6 months, and 12 months postoperatively [33]. Like our study and in contrast to Retrouvey’s study, psychosocial well-being declined slightly at the 6-month postoperative time point following BCS and MIBR. In both of these prior studies, as well as ours, TMNR reported the largest decline and lowest scores postoperatively.

Based on the trends observed in our study, patients with both MIBR and TMNR appear to have poorer HRQoL outcomes compared to BCS. This has also been found in other studies that have assessed postoperative results [29]. In our study, breast satisfaction remained stable after BCS and decreased after MIBR. Although psychosocial well-being scores are similar pre- and postoperatively in both our BCS and MIBR groups, the absolute scores are better in the BCS group. This is similar to Retrouvey’s findings but in contrast to Devarakonda’s findings. We found a larger decrease in scores postoperatively in the MIBR group than Retrouvey, with the overall decrease in our MIBR and TMNR groups being similar, whereas the results were comparable in the TMNR group between our and Retrouvey’s studies. Additionally, our study indicated that MIBR patients experienced the highest postoperative anxiety and pain, which was not seen in Retrouvey’s study. The difference between our results and those of Retrouvey and Devakondra may be in part due to the larger number of BCS patients in our study, with our study showing a proportion of BCS reflective of our overall practice. Another factor may be that all patients at our center are offered consultation with a plastic surgeon, so we had fewer patients with TMNR who wanted reconstruction, which could affect the results.

In this study, we also identified that younger age was an independent predictor for higher postoperative depression, pain, and anxiety scores, and adjuvant chemotherapy was associated with higher postoperative depression scores. Neither Retrouvey’s nor Devarakonda’s studies investigated these relationships. However, it has been well demonstrated in the literature that younger age is associated with an increased risk of developing depression and anxiety following a cancer diagnosis. A study assessing factors mediating persistent postsurgical pain in younger breast cancer patients identified that younger age was associated with greater depression, anxiety, and sleep disturbances postoperatively, in turn likely contributing to greater chronic pain-related functional disability among younger patients [34]. A retrospective study assessing risk factors for postoperative depression in breast cancer patients identified younger age as a significant risk factor [35]. This increased risk may be due to persistent cancer-related fears, including concerns about cancer recurrence in particular [36].

Other studies have had variable results with regard to the effect of chemotherapy on postoperative psychosocial well-being and depression. Christensen et al. found an association of depression with treatment with chemotherapy [37]. However, Flanagan et al. [29] did not find an association with chemotherapy and psychosocial well-being, but it was not divided into adjuvant and neoadjuvant chemotherapy as in our study. The association between depression and cancer treatment is well established, with it recognized that depression is more prevalent in breast cancer than in other tumor types [38]. Patients undergoing chemotherapy may have more advanced or high-risk cancers, which may account for this finding. Alternatively, seeing that neoadjuvant chemotherapy did not have a significant association, whereas adjuvant chemotherapy did, it is possible that this reflects closer timing to chemotherapy treatment at the time of the postoperative survey, and that with further time from treatment, this association may diminish. Clinical guidelines recognize the prevalence and importance of psychological distress in breast cancer patients, and it is recommended that patients be routinely screened and offered pharmacologic and non-pharmacologic supports in addition to traditional care [39]. Our findings further support the practice of regular distress screening during treatment.

Although radiation use was different across surgical modalities, it was not associated with significant differences in postoperative pain, depression, or anxiety. The higher use of radiation post-BCS was expected, but it is noteworthy that patients post-mastectomy had radiation nearly half of the time (48.33% TMNR, 42.86% MIBR). Post-mastectomy radiotherapy is known to impact the reconstructed breast and may result in capsular contracture or implant loss [40]. It has also been associated with worse breast satisfaction over time in BCS and MIBR [29]. Although patients wanting to avoid radiation tend to opt for mastectomy, with modern treatment guidelines, patients should be informed that there is a high likelihood of recommendations for radiation regardless of the surgical approach.

Our study had some limitations. First, it was not sufficiently powered to account for differences in patient-specific tumor characteristics, as well as other clinical factors, such as treatment complications, which may influence a patient’s 6-month follow-up HRQoL reported. Second, whereas both Retrouvey et al. and Devarakonda et al. had preoperative, 6-month, and 12-month postoperative follow-up time points, our study did not have the 12-month time point. Although this shortened follow up time is a limitation of our study as some participants may still be in the process of receiving adjuvant treatments at 6 months, the 12-month BREAST-Q scores reported by both Retrouvey et al. and Devarakonda et al. did not differ considerably from the 6-month time point, and the overall trend of the BREAST-Q scores (increasing or decreasing from baseline preoperative) did not change from the 6-month to 12-month time point. Lastly, we acknowledge that the COVID-19 (coronavirus disease 2019) pandemic added unprecedented external stressors for patients unrelated to their breast cancer surgery experience that may influence the reported HRQoL outcomes. The majority of participants in our study were treated before the declaration of the global pandemic and lockdown, which minimizes the impact of any pandemic-related stressors on the HRQoL outcomes reported.

Our finding of different results when comparing postoperative PROMs alone versus the change in pre- to postoperative PROMs is relevant to clinical practice and future research. This study has allowed us to update our information to discuss with patients at surgical consultation for the selection of surgical treatment. Additionally, understanding the effect of preoperative and change in PROMs on postoperative outcomes is an opportunity for additional support at the preoperative time point, and we are working on adding mental health supports preoperatively, as previously, distress screening occurred at the time of consultation at the cancer center, which was usually postoperative. Our findings underline the importance of being able to compare pre- and postoperative scores in future studies. As we identified younger age to be an independent predictor for postoperative depression and anxiety, future areas of research should focus on comparing the HRQoL outcomes between younger and older breast cancer surgery patients to identify specific differences in the various PROMs, as significant differences may indicate distinct surgical treatment priorities and expectations between the groups. Based on the findings of this research, we are developing clinical programs to use this data to enhance support for patients with a particular focus on the needs of younger patients.

## 5. Conclusions

Our study reveals that BCS may have better overall HRQoL outcomes, specifically in breast satisfaction, psychosocial, physical, and sexual well-being, compared to TMNR and MIBR. Additionally, multivariable regression analysis identified that younger age, rather than surgical modality, was associated with higher postoperative depression, pain, and anxiety scores, and adjuvant chemotherapy was associated with higher postoperative depression scores. These differences in HRQoL outcomes are important to consider in the context of breast cancer survivorship and should be discussed with patients during surgical consultation to ensure comprehensive discussions about mental health supports, setting expectations, and enhancing shared patient decision making. Tailoring surgical choices to individual patient profiles can optimize oncologic, surgical, and HRQoL outcomes, particularly in the context of breast reconstruction counseling. This approach ensures each patient receives the most appropriate surgery, enhancing survivorship and quality of life.

## Figures and Tables

**Table 1:** Distribution of baseline covariates by surgical modality of breast-conserving surgery, total mastectomy with no reconstruction, and total mastectomy with immediate breast reconstruction. *p*-values were reported compared to distribution in each stratum of the covariates across the surgical modalities.

			Total Mastectomy (N = 157/32.18%)		
Patient Characteristic	Overall Sample	Breast-Conserving Surgery (BCS)	No Reconstruction (TMNR)	Immediate Reconstruction (MIBR)	
	(N = 471)	(N = 313/66.45%)	(N = 60)	(N = 98)	*p*-Value
Age Category					<0.01
≤49	131 (27.81%)	67 (21.41%)	10 (16.67%)	54 (55.10%)	
50–59	107 (22.72%)	65 (20.77%)	15 (25.00%)	27 (27.55%)	
60–69	131 (27.81%)	106 (33.87%)	15 (25.00%)	10 (10.20%)	
≥70	102 (21.66%)	75 (23.96%)	20 (33.33%)	7 (7.14%)	
Charlson Index					<0.01
0	75 (15.92%)	52 (16.61%)	3 (5.00%)	20 (20.41%)	
1–2	265 (56.26%)	195 (62.30%)	23 (38.33%)	47 (47.96%)	
3+	126 (26.75%)	63 (20.13%)	34 (56.66%)	29 (29.59)	
SES Income—Situational vulnerability					0.77
Q1 Least Vulnerable	132 (28.03%)	88 (28.12%)	13 (21.67%)	31 (31.63%)	
Q2	102 (21.66%)	67 (21.71%)	12 (20.00%)	23 (23.47%)	
Q3	92 (19.53%)	59 (18.84%)	15 (25.00%)	18 (18.37%)	
Q4	99 (21.02%)	67 (21.41%)	12 (20.00%)	20 (20.41%)	
Q5 Most Vulnerable	40 (8.49%)	28 (8.95%)	7 (11.67%)	5 (5.10%)	
SES—Ethno-cultural composition					0.17
Q1 Least Diverse	14 (2.97%)	8 (2.56%)	2 (3.33%)	4 (4.08%)	
Q2	24 (5.10%)	18 (5.75%)	2 (3.33%)	4 (4.08%)	
Q3	81 (17.20%)	64 (20.45%)	4 (6.67%)	13 (13.27%)	
Q4	182 (38.64%)	119 (38.02%)	23 (38.33%)	40 (40.82%)	
Q5 Most Diverse	164 (34.82%)	100 (31.95%)	28 (46.67%)	36 (36.73%)	
Axillary Surgery					<0.01
None	83 (17.62%)	77 (24.60%)	2 (3.33%)	4 (4.08%)	
Sentinel Node Biopsy	341 (72.40%)	223 (71.25%)	36 (60.00%)	82 (83.67%)	
Axillary Dissection	47 (10.00%)	13 (4.15%)	22 (36.67%)	12 (12.24%)	
Neoadjuvant Chemotherapy					<0.01
No	413 (87.69%)	288 (92.01%)	45 (75.00%)	80 (81.63%)	
Yes	58 (12.31%)	25 (7.99%)	15 (25.00%)	18 (18.37%)	
Adjuvant Chemotherapy					<0.01
No	339 (71.97%)	246 (78.59%)	38 (63.33%)	55 (56.12%)	
Yes	132 (28.03%)	67 (21.41%)	22 (36.67%)	43 (43.88%)	
Hormone Therapy					0.63
No	165 (35.03%)	108 (34.50%)	19 (31.67%)	38 (38.78%)	
Yes	305 (82.21%)	204 (65.18%)	41 (68.33%)	60 (61.22%)	
Aduvant Radiation					<0.01
No	141 (29.94%)	54 (17.25%)	31 (51.67%)	56 (57.14%)	
Yes	329 (69.85%)	258 (82.43%)	29 (48.33%)	42 (42.86%)	

**Table 2:** Summary of multivariable linear regression analysis of covariance model for postoperative PHQ-9 depression score and GAD-7 anxiety score.

	PHQ-9			GAD-7		
Regression Effect	Estimate	Standard Error	*p*-Value	Estimate	Standard Error	*p*-Value
Intercept	9.13	1.13	<0.01	8.67	1.12	<0.01
Age (Years)	−0.05	0.02	<0.01	−0.08	0.01	<0.01
Surgery						
Breast-Conserving Surgery	Reference			Reference		
Mast—No Reconstruction	−0.22	0.65	0.72	−0.06	0.68	0.92
Mast—Immediate Reconstruction	−0.11	0.55	0.83	0.37	0.54	0.48
Situational vulnerability						
Q1 Least Vulnerable	−0.37	0.56	0.50	−1.08	0.57	0.05
Q2	−0.32	0.55	0.55	−0.54	0.56	0.33
Q3	0.01	0.54	0.98	−0.75	0.54	0.17
Q4 + Q5 Most Vulnerable	Reference			Reference		
Ethno-cultural composition						
Q1 + Q2 + Q3 Least Diverse	0.05	0.56	0.92	0.09	0.58	0.87
Q4	0.34	0.48	0.47	0.65	0.49	0.17
Q5 Most Diverse	Reference			Reference		
Charlson Index						
0	−0.18	0.75	0.80	−0.33	0.75	0.66
1–2	−0.27	0.47	0.56	−0.46	0.48	0.34
3+	Reference			Reference		
Neoadjuvant Chemotherapy						
No	−1.14	0.63	0.07	−0.37	0.63	0.58
Yes	Reference			Reference		
Adjuvant Chemotherapy						
No	−1.25	0.47	<0.01	0.02	0.48	0.95
Yes	Reference			Reference		
Hormone Therapy						
No	−0.36	0.45	0.41	−0.10	0.45	0.81
Yes	Reference			Reference		
Adjuvant Radiation						
No	0.29	0.49	0.54	−0.18	0.48	0.70
Yes	Reference			Reference		

## Data Availability

Requests for anonymized data presented in this study can be directed to Jason M. Sutherland.

## Abbreviations

HRQoL	Postoperative health-related quality of life.
BCS	Breast-conserving surgery.
TMNR	Total mastectomy no reconstruction.
MIBR	Total mastectomy immediate breast reconstruction.
PROM	Patient-reported outcome measures.
SES	Socioeconomic status.
CIMD	Canadian Index of Multiple Deprivation.
CCI	Charlson Comorbidity Index.
HADS	Hospital Anxiety and Depression Scale.

## Appendix A

**Table A1:** Summary of preoperative and 6-month postoperative patient-reported outcomes for the overall sample and stratified by breast-conserving surgery and total mastectomy with immediate (MIBR) or no reconstruction (TMNR). Values in table are reported as mean score (standard deviation). *p*-values are reported for comparison of mean change in score values between breast-conserving surgery, total mastectomy with no reconstruction, and total mastectomy with immediate reconstruction groups.

					Total Mastectomy				
	Overall Sample		Breast-Conserving Surgery (BCS)		No Reconstruction (TMNR)		Immediate Reconstruction (MIBR)		
Patient-Reported Outcome Measure	Preoperative	Postoperative	Preoperative	Postoperative	Preoperative	Postoperative	Preoperative	Postoperative	*p*-Value
**Breast-Q**									
Satisfaction with Breast	66.24 (22.16)	63.19 (23.31)	68.57 (21.68)	67.22 (23.44)	57.24 (24.41)	49.64 (20.40)	63.29 (20.55)	56.96 (19.17)	0.21
Psychosocial well-being	72.80 (20.16)	71.80 (21.14)	74.23 (19.59)	74.38 (21.24)	70.22 (22.48)	63.71 (19.97)	68.75 (20.21)	67.34 (20.23)	0.10
Physical well-being chest	78.75 (15.83)	73.98 (16.59)	80.46 (15.63)	76.22 (16.35)	74.37 (16.69)	68.31 (16.58)	74.99 (14.89)	68.61 (15.46)	0.42
Sexual well-being	57.50 (24.61)	50.34 (26.06)	58.60 (24.43)	52.92 (24.77)	49.21 (26.70)	39.80 (30.50)	58.23 (23.50)	47.58 (26.12)	0.76
PHQ-9	4.40 (4.50)	4.07 (4.20)	4.15 (4.46)	3.84 (4.09)	3.72 (3.82)	4.10 (4.43)	5.60 (4.80)	4.78 (4.39)	0.15
GAD-7	4.39 (4.70)	3.11 (3.75)	3.85 (4.27)	2.94 (3.68)	4.00 (4.33)	2.39 (3.08)	6.36 (5.71)	3.93 (4.13)	0.03
PEG	1.51 (2.14)	1.83 (2.27)	1.43 (2.10)	1.65 (2.25)	1.96 (2.51)	1.98 (2.21)	1.50 (2.04)	2.29 (2.33)	0.05
EQ-5D-5L (VAS)	73.56 (19.18)	76.76 (15.76)	74.41 (18.90)	77.69 (15.75)	67.41 (22.72)	72.64 (16.60)	74.64 (17.10)	76.41 (15.04)	0.48

**Table A2:** Summary of postoperative patient-reported outcomes for the overall sample and stratified by breast-conserving surgery and total mastectomy with immediate (MIBR) or no reconstruction (TMNR). Values in table are reported as mean score (standard deviation). *p*-values are reported for comparison of postoperative score values between breast-conserving surgery, total mastectomy with no reconstruction, and total mastectomy with immediate reconstruction groups.

	Postoperative Scores				
Patient-Reported Outcome Measure	Overall Sample	Breast-Conserving Surgery (BCS)	No Reconstruction (TMNR)	Immediate Reconstruction (MIBR)	*p*-Value
**Breast-Q**					
Satisfaction with Breast	63.19 (23.31)	67.22 (23.44)	49.64 (20.40)	56.96 (19.17)	<0.01
Psychosocial well-being	71.80 (21.14)	74.38 (21.24)	63.71 (19.97)	67.34 (20.23)	<0.01
Physical well-being chest	73.98 (16.59)	76.22 (16.35)	68.31 (16.58)	68.61 (15.46)	<0.01
Sexual well-being	50.34 (26.06)	52.92 (24.77)	39.80 (30.50)	47.58 (26.12)	<0.01
PHQ-9	4.07 (4.20)	3.84 (4.09)	4.10 (4.43)	4.78 (4.39)	0.15
GAD-7	3.11 (3.75)	2.94 (3.68)	2.39 (3.08)	3.93 (4.13)	0.05
PEG	1.83 (2.27)	1.65 (2.25)	1.98 (2.21)	2.29 (2.33)	0.04
EQ-5D-5L (VAS)	76.76 (15.76)	77.69 (15.75)	72.64 (16.60)	76.41 (15.04)	0.07

## Appendix B

**Table A3:** Summary of multivariable linear regression analysis of covariance model for postoperative PEG score.

	PHQ-9		
Regression Effect	Estimate	Standard Error	*p*-Value
Intercept	4.61	0.67	<0.001
Age (Years)	−0.024	0.01	0.01
Surgery			
Breast-Conserving Surgery	Reference		
Mast—No Reconstruction	0.05	0.37	0.90
Mast—Immediate Reconstruction	0.25	0.32	0.43
Situational vulnerability			
Q1 Least Vulnerable	−0.57	0.31	0.06
Q2	−0.75	0.30	0.01
Q3	−0.36	0.30	0.23
Q4 + Q5 Most Vulnerable	Reference		
Ethno-cultural composition			
Q1 + Q2 + Q3 Least Diverse	0.36	0.31	0.24
Q4	0.06	0.27	0.81
Q5 Most Diverse	Reference		
Charlson Index			
0	−0.56	0.46	0.23
1–2	−0.47	0.28	0.09
3+	Reference		
Neoadjuvant Chemotherapy			
No	−0.37	0.38	0.33
Yes	Reference		
Adjuvant Chemotherapy			
No	−0.47	0.26	0.07
Yes	Reference		
Hormone Therapy			
No	0.16	0.25	0.51
Yes	Reference		
Adjuvant Radiation			
No	−0.09	0.27	0.74
Yes	Reference		
Axillary Surgery			
None	−0.35	0.56	0.53
Sentinel Node Biopsy	−0.14	0.44	0.74
Axillary Dissection	Reference		
